# Microbial quorum sensing signals, symbiosis, secrets and sabotage

**DOI:** 10.1099/mic.0.001752

**Published:** 2026-07-29

**Authors:** Miguel Cámara, George P.C. Salmond, Paul Williams

**Affiliations:** 1 National Biofilms Innovation Centre, Biodiscovery Institute, School of Life Sciences, University of Nottingham, Nottingham NG7 2RD, UK; 2 Department of Biochemistry, University of Cambridge, Cambridge, CB2 1QW, UK

**Keywords:** autoinducer, cell-cell signalling, intercellular signalling, polymicrobial interactions, quorum quenching, quorum sensing

## Abstract

Bacteria exhibit sophisticated forms of multicellularity by coordinating the activities of individual cells with respect to behaviours such as symbiosis, conjugation, competence, swarming, secondary metabolite production, biofilm formation, growth inhibition, niche adaptation and virulence. Apart from direct cell–cell contact, the synthesis, release and perception of small diffusible chemicals acting as inter-cellular signals offer the most obvious mechanism for bacterial cell populations to synchronize their behaviour. Such cell–cell communication (quorum sensing) offers a unique framework for understanding how bacteria convert chemical information into coordinated group behaviours. It is deeply influenced by ecology, host interactions, chemical diversity and spatial architecture. Despite much progress, many fundamental questions remain unresolved, especially in natural, polymicrobial and host-associated environments. Here, we summarize our current understanding of bacterial cell–cell communication and main knowledge gaps and highlight examples of translational opportunities.

## Introduction

### Discovery and early insights

Hints that cell–cell communication existed in bacteria first emerged over 60 years ago when it was proposed that the transient and synchronous expression of competence in pneumococcal populations was controlled by extracellular, cell-produced factors, implying that a bacterial population could temporarily behave as a coordinated biological unit [1]. A further early example came from studies of the *γ*-butyrolactone A-factor from *Streptomyces griseus*, which was shown to control streptomycin biosynthesis and aerial mycelium formation [2]. Soon after, the term ‘autoinduction’ was introduced as a mechanism of controlling bioluminescence in marine vibrios [3]. Widespread acceptance of the bioluminescence autoinduction model came with the chemical characterization of the *Aliivibrio fischeri* (formerly *Vibrio fischeri*) autoinducer as *N*-(3-oxohexanoyl)-l-homoserine lactone and the identification of the *luxICDABE* operon and adjacent *luxR* gene in this organism [4, 5]. Subsequent studies revealed that many different non-bioluminescent terrestrial Gram-negative bacteria produced *N*-acylhomoserine lactones (AHLs) and possessed orthologues of the AHL synthase (LuxI) and AHL response regulator (LuxR) [6, 7]. With respect to these LuxRI-based autoinduction systems, the term ‘quorum sensing’ (QS) was introduced to define behaviours that can be performed efficiently only by a sufficiently large population of bacteria where the minimal unit is the ‘quorum of bacteria’ [6].

These advances in our understanding of cell–cell communication in bacteria raised many questions relating to the molecular diversity and specificity of AHLs, the structure/function and the activation/inhibition profiles of LuxI and LuxR proteins given their low homologies and their substrates and selectivity with respect to AHL biosynthesis and LuxR receptor recognition, as well as the identity of their downstream target genes. It also emerged that many bacteria, including *V. fischeri*, employ multiple LuxRI/AHL systems that are integrated with each other and are also linked to non-AHL systems such as 2-alkyl-4-quinolone (AQ) signalling in *Pseudomonas aeruginosa*. Genomics, transcriptomics and proteomics have aided the rapid identification of the vast repertoire of target genes regulated by LuxRI/AHL systems. While not all bacterial species make AHLs, parallel advances in our understanding of non-AHL-based QS have cemented the universality of the QS paradigm throughout the microbial world (including bacteria, fungi, protozoa and algae). The QS paradigm also extends beyond microbial–microbial conversations: analogous quorum-like threshold decision processes have been described in social insects such as ants and honeybees, while bacterial QS molecules can be perceived by eukaryotic hosts, including plants and mammalian cells, where they alter defence, stress or apoptotic signalling pathways [8–11].

### LuxRI/AHL system diversity

Most AHL producers synthesize multiple AHLs, which are characterized by a homoserine lactone ring unsubstituted in the *β*- and *γ*-positions, which is *N*-acylated with a fatty acyl group at the *α*-position [12]. The acyl chain varies in length (C_4_-C_20_), saturation level and oxidation state with most AHLs belonging either to the *N*-acyl, *N*-(3-oxoacyl) or *N*-(3-hydroxyacyl) classes, although *N*-aryl AHLs [*N*-(p-coumaroyl)-l-HSL and *N*-cinnamoyl-HSL] and the branched-chain *N*-isovaleryl-l-HSL have been isolated [13, 14]. The shortest acyl-chain AHLs encountered are C4-acyl chains, likely because the HSL ring is highly susceptible to pH-dependent ring opening, which decreases as the acyl side chain is lengthened [15]. Interestingly, while *N*-carboxylated AHLs are produced via a LuxI orthologue in methanogenic archaea [16], other non-AHL synthases have also been described. Curiously, most AHL synthases drive the synthesis of multiple AHLs, not all of which can activate the endogenous LuxR protein, implying that AHLs have other biological functions.

In the post-genomic era, the vast number of bacterial genome sequences interrogated have revealed that, where present, *luxR* and *luxI* genes are frequently genetically linked either on the chromosome or on mobile genetic elements but may be arranged in different topologies (i.e. convergent, divergent or in tandem) or separated by a negative regulatory gene [17]. In addition to harbouring more than one *luxRI* pair, many bacteria also possess one or more unpaired but closely related *luxR* genes. The products of the latter are termed LuxR ‘orphans’ or ‘solos’ [17]. LuxR proteins are mostly transcriptional activators that bind AHLs with a 1 : 1 stoichiometric ratio of protein to AHL. AHL binding induces dimerization and a conformational change, enabling the LuxR/AHL complex to bind ‘*lux*’ boxes located in the promoter regions of target genes. Although LuxR homologues display low primary sequence similarities (18%–25%), the structures of both domains display nine highly conserved amino acid residues that constitute the AHL-binding site. LuxR solos may respond to endogenous or exogenous AHLs, function in a ligand-independent manner or bind non-AHL signals generated endogenously or exogenously [17]. For example, *Escherichia coli* and *Salmonella* do not produce AHLs but possess a LuxR homologue called SdiA that responds to exogenously produced AHLs, while CarR, a LuxR homologue that regulates carbapenem antibiotic production in *Serratia*, is ligand-independent [17]. Most LuxRs are highly selective for their cognate endogenous AHL, while others demonstrate receptor promiscuity and cross-species eavesdropping and can bind multiple AHLs that structurally differ only slightly [18, 19]. Therefore, cross-talk appears to be an intrinsic property, rather than an exception. Why many bacteria retain multiple LuxRI systems in a single strain (e.g. *Rhizobium* strains may have up to seven systems [20]) is not obvious. One plausible explanation is that chemically distinct signals with different stabilities, diffusion behaviours or regulatory connectivities may allow cells to integrate both social information (population density or relatedness) and physical information (mass transfer, confinement or flow) from their environment [21].

### QS beyond the AHLs

Apart from the AHLs and the closely related *N*-aryl-homoserine lactones, diverse chemical classes of QS signal molecules found in Gram-negatives include the AQs, diketopiperazines, cis-2-unsaturated fatty acids, 3-hydroxy-fatty acid methyl esters, *α*-hydroxyketones, pyrazinones, *α*-pyrones and dialkylresorcinols [22]. Recently, Linares-Otoya *et al.* [23] discovered a family of *N*-acyl-cyclolysines (ACLs) in the phylum Bacteroidota and reported that the ACL system is widely distributed in both human gut and oral microbiome samples. The furanosyl boronate ester autoinducer-2 (AI-2), synthesized via LuxS, is found in both Gram-positive and Gram-negative bacteria [24]. Depending on the species, AI-2 can function as a QS signal, a chemical cue or a metabolic waste product [25, 26]. While Gram-positive bacterial species do not appear to produce AHLs, they commonly employ linear, modified or cyclic peptides or γ-butyrolactones as QS signal molecules [27–30]. QS-type responses have also been identified in archaea, although the nature of the signals remains unknown [31].

### QS within ecosystems

QS not only constitutes cell–cell communication within a single bacterial species but also shapes social networks given that bacteria cohabit many different ecosystems with other bacterial species and organisms from different kingdoms [32]. In soils and the rhizosphere, steep chemical gradients and spatial structures at the root–soil interface promote local signal accumulation and densely interacting microbial assemblages, helping make QS ecologically relevant *in situ*. In this environment, QS-regulated behaviours such as colonization, biofilm formation and the production of secondary metabolites and antimicrobials can reshape microbe–microbe interactions [33]. Plants not only ‘listen in’, as bacterial AHLs can alter plant physiology and prime defence responses [34], but they can also release small molecules that interfere with bacterial QS (including signal mimics or inhibitory activities), providing a route for host control over bacterial collective behaviours within the root zone [35]. Conversely, plant-derived metabolites can be detected by subfamilies of LuxR solos in plant-associated bacteria, directly linking host chemistry to bacterial transcriptional programmes in the rhizosphere and endosphere [17].

Signalling disruption may also arise through enzymatic inactivation (quorum quenching) and sequestration of QS signal molecules, by producing QS signal molecule mimics or by deploying QS pathway inhibitors. For example, zoospores of the biofouling marine macroalga *Ulva* utilize bacterial AHLs as cues for the selection of inter-tidal sites for surface attachment and subsequent morphological development [36]. However, AHL-degrading bacterial strains in marine communities manipulate zoospore settlement by interfering with QS between neighbouring bacteria [32]. AHLs also mediate the settlement of polychaete larvae, bryozoans and barnacles [37–39]. The red alga, *Delisea pulchra*, produces halogenated furanones that interfere with AHL-dependent QS pathways [40]. By targeting QS in *Chromobacterium violaceum* with an AHL-inactivating lactonase, major changes in social interactions between this organism, Gram-positive bacteria, yeast, macrophages and flatworms have been observed [41].

Thus, in natural environments where QS is rarely independent of higher organisms, a number of bidirectional interactions have been identified, suggesting that host environments impose selective pressures that shape QS network evolution [38, 42]. Furthermore, QS signal molecules produced by *P. aeruginosa* possess concentration-dependent modulatory properties that impact on mammalian immune and cardiovascular systems [43]. In the gastro-intestinal tract, the microbiota and host have co-evolved into a symbiotic relationship in which the bacteria provide the host with additional metabolites and metabolic functions and promote intestinal tract development, immune system maturation and protection against invading pathogens. Evidence that diverse QS signalling systems help shape a healthy gut is just beginning to emerge [44], as is the role of host molecules in activating or inhibiting QS. For example, skin androgens such as testosterone regulate *Staphylococcus aureus* pathogenicity independently of the cognate staphylococcal autoinducing peptide (AIP) by directly activating the AIP receptor, AgrC [45].

## Unanswered questions and considerations

**Fig. 1.**
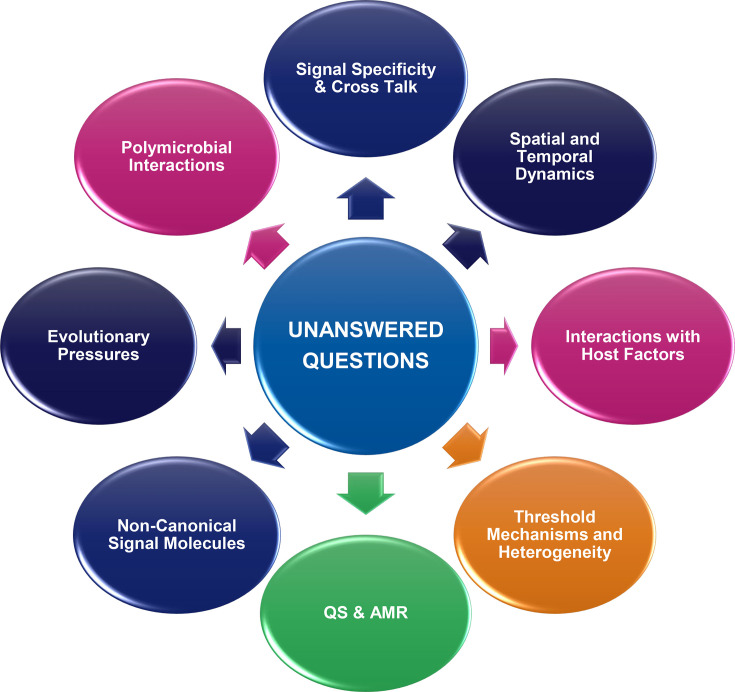
Major unresolved questions in QS research. The schematic summarizes the principal knowledge gaps discussed in this Perspective. These include the need to redefine QS beyond a simple “quorum” concept; to quantify signalling thresholds and heterogeneity in spatially structured and host-associated environments; to identify non-canonical signal molecules and receptors; to understand signalling interactions in polymicrobial communities; to resolve the links between QS, antimicrobial tolerance, and host modulation; and to determine how ecological and evolutionary pressures shape the robustness, plasticity, and therapeutic tractability of QS networks.

A summary of the unanswered questions in the QS field is provided in Fig. 1.

### QS beyond the ‘quorum’

The term ‘QS’ as originally defined by Fuqua *et al*. [6] does not adequately describe all situations where bacteria employ diffusible chemical signals. It has become clear that the size of the ‘quorum’ is not fixed at a high population density but varies according to local environmental conditions. QS is one of many different environmental parameters (e.g. temperature, pH, osmolarity, oxidative stress and nutrient deprivation) that bacteria must integrate via global gene regulatory networks to determine their optimal survival strategy [46]. It is also clear that an individual bacterial cell can switch from the ‘non-quorate’ to the ‘quorate’ state as shown for a single bacterium trapped within an endosome in endothelial cells [47]. In essence, QS is an environmental sensing mechanism that titrates the concentration of a diffusible extracellular signal molecule. Activation or repression of target genes occurs when a critical threshold signal molecule concentration (not cell number) is reached. Becoming quorate depends on the relative rates of production and loss of a diffusible signal. Therefore, the original question with regard to ‘What is the QS activation concentration?’ has shifted to ‘What is the threshold concentration for activation within a specific environment?’ as it is recognized that thresholds will differ between individual cells and micro-environments. This view also provides a concise framework for understanding why some bacteria retain multiple QS circuits: rather than representing simple redundancy, distinct signals may differ in diffusion, decay, receptor affinity, regulatory timing and sensitivity to mass transfer, allowing combinatorial responses that help cells distinguish local population density from physical constraints such as confinement, flow or diffusion limitation [21].

Classic QS models support a threshold concentration leading to synchronous population behaviour. However, single-cell studies revealed substantial heterogeneity, with subpopulations activating QS early, late or not at all [48]. This heterogeneity may be advantageous, as it may function as a bet-hedging strategy to ensure survival under fluctuating environmental conditions. How such heterogeneous subpopulations collectively influence virulence, biofilm formation and antibiotic tolerance remains to be understood. Moreover, QS does not invariably coordinate a social public good phenotype: in young *Pseudomonas putida* biofilms, AHL production is stochastic and triggers putisolvin-dependent escape of individual cells from microcolonies, illustrating that QS can regulate asocial, self-directed motility, as well as cooperative group behaviours [49].

Care must also be taken in defining a molecule as a QS signal since such a signal should benefit both receiver and producer cells. This is because the molecule may be simply acting as a chemical cue by the receiver [25, 50]. To be classed as a QS signal, a candidate signal should display characteristics such as the following: (1) production should take place during specific stages of growth or in response to specific environmental challenges; (2) accumulation in the extracellular environment and recognition by a specific bacterial receptor; (3) attainment of a threshold concentration should generate a concerted physiological response; and (4) the cellular response should extend beyond the physiological changes required to metabolize or detoxify the putative QS signal molecule. QS raises many questions with respect to social evolution, fitness and the benefits associated with costly co-operative behaviours [25, 51]. Some of these have now been addressed experimentally, demonstrating that QS, at least, in certain laboratory environments is indeed social, regulating the production of extracellular public goods and vulnerable to cheating by QS mutants which gain benefits without having to maintain an energetically costly functional QS system [26, 52].

### Spatial and temporal dynamics of QS activation

The foundational studies on QS were performed in homogenous laboratory cultures, supporting the classical idea of QS as a uniform, population-wide switch. However, it is now known that the spatial structure of microbial communities dramatically alters QS behaviour [51]. Biofilms create steep chemical gradients, and the flow of fluid redistributes signals, making the signalling activation and repression highly heterogeneous [53, 54]. It is known that, *in vivo*, signals accumulate in microniches resulting in patchy and transient QS activation patterns [55].

The diffusion barriers encountered in biofilms and host tissues result in adjacent microcolonies potentially experiencing considerably different signal concentrations. These spatial effects pose a challenge to the concept of a simple quorum threshold, supporting the idea that QS operates in a spatiotemporal manner. However, technical challenges to accurately map QS gradients *in vivo* are hindering progress in this area. Although real-time biosensors, imaging mass spectrometry and microfluidic infection models have begun to bridge this gap, the precise quantification of *in vivo* QS concentrations remains an unresolved challenge.

### Non-canonical QS molecules and receptors

Non-canonical QS molecules are considered those endogenous or exogenous small molecules that influence population-density-dependent behaviours by engaging QS regulatory networks (directly or indirectly), while not belonging to the established AI families and/or not being produced primarily via dedicated QS synthases. These QS molecules likely far outnumber those currently characterized. In the ocean, a deep-sea cold-seep metagenomic survey found 299,355 QS-related genes across 12 archaeal and 108 bacterial phyla, implying many undiscovered signals [56]. In soils/rhizospheres, genome surveys show that LuxR solos are far more common than complete LuxI/LuxR pairs and often carry altered ligand-binding pockets, suggesting that they are involved in the detection of non-AHL ligands which remain to be chemically defined [17, 57]. In plant-associated settings, the difussible signal factor, known as DSF, regulates traits in pathogens and biocontrol bacteria and can even modulate plant immunity, reinforcing the likelihood that many rhizosphere signals and receptors remain uncharacterized [58]. However, the complete repertoire of QS signal molecules remains largely unknown. To further our understanding of their true overall impact, signals must be contextualized, linked to the environments in which they emerge and to the community and host processes they modulate. This could be accelerated by scaling up high-throughput biosensor platforms and high-resolution tandem mass spectrometry tuned to these chemistries, allowing direct surveillance of their presence and activity in natural settings.

### QS in communities

One of the most recent transformative shifts in microbiology has been the recognition that bacteria nearly always exist in multi-species communities. Yet the ‘QS interaction networks’ of polymicrobial communities remain almost completely unmapped. Single-species models cannot capture the complexity of cross-species interference, synergistic activation, competitive inhibition or shared public goods [59, 60]. In polymicrobial environments, where many species coexist, the challenge of maintaining communication fidelity is hugely increased. In mixed-species infections, bacteria frequently exploit signal overlap to influence the behaviour of competitors or to detect community composition [61]. However, the ecological consequences of such cross-signalling remain poorly understood, including whether signalling interference can override species-specific communication and how much ‘noise’ bacteria are able to tolerate. Although newer analytical techniques have begun dissecting these interactions, definitive answers are still lacking. Untangling this bidirectional, interkingdom communication remains a major challenge, particularly the consequences of such interactions for both health and disease.

Future work needs to move from cataloguing signals to mapping who produces, receives, modifies and degrades them across defined community structures. In biofilms, wounds, cystic fibrosis airways, dental plaque, soil aggregates and rhizospheres, QS molecules can act as shared currencies, private messages, public goods, competitive weapons, developmental cues or host-facing metabolites. Community composition can therefore alter both the meaning and the outcome of a signal: the same molecule may activate cooperation among kin, be intercepted by competitors, be degraded by quorum-quenching neighbours or be sensed by eukaryotic hosts. Spatial organization adds another layer, because microniches can create local quora even when the bulk community appears dilute. A major unresolved challenge is to combine metagenomics, metabolomics, spatial transcriptomics, biosensors and experimentally tractable synthetic communities to determine how QS networks influence community behaviour.

### QS and antimicrobial tolerance

QS plays a central role in antimicrobial tolerance through the regulation of biofilm formation, extracellular matrix production, stress survival pathways, efflux pump expression and multispecies metabolic cooperation, contributing to persistent infections and treatment failure. Moreover, QS influences interspecies signalling, enabling polymicrobial communities to behave as integrated, highly tolerant units that resist antibiotic penetration and clearance. This is aggravated by sub-inhibitory antibiotic concentrations unintentionally stimulating QS networks, reshaping microbial community behaviour and elevating resistance in multispecies settings such as chronic wounds or cystic fibrosis lung infections. Given these intertwined mechanisms, future research must prioritize disentangling antibiotic-induced QS activation, resolving how QS contributes to polymicrobial antimicrobial tolerance and identifying which QS-regulated pathways represent the most actionable therapeutic targets [62–64].

### Evolutionary pressures

The evolution of QS remains a conceptual challenge. Cheaters, non-producers that exploit public goods, erode QS cooperation. However, QS is widespread, and it remains to be understood why it is evolutionarily stable. Early evolutionary studies invoked kin selection, policing mechanisms and spatial structure [50]. Subsequent research suggests that polymicrobial environments, host-immune interactions and antimicrobial pressures strongly shape QS evolution [63]. However, the extent to which these pressures drive signalling networks to rapidly mutate and rewire (as observed, for example, with the *S. aureus agr* QS system [30]), enabling microbes to escape from host interference or therapeutic interventions, remains unclear. Whether this process can be reversed *in vivo* also remains to be established. The evolutionary adaptability of QS means that any consideration of therapeutically targeting it should anticipate the consequences of long-term microbial evolution.

### Translational potential

As knowledge of QS has advanced, opportunities to exploit or disrupt bacterial cell–cell communication systems have emerged. Synthetic biologists have constructed or rewired genetic circuitry responsive to population density in order to engineer synthetic consortia with improved temporal and spatial control of gene expression or to increase product yields or to drive pattern formation or even to facilitate communication with abiotic materials to create hybrid microelectronic devices [65, 66]. Manipulating QS within microbial communities, which form the core of bio-electrochemical systems, holds promise for sustainable energy generation and wastewater treatment [67]. AHLs and AHL-signalling plant-beneficial bacteria are important elements in bacteria–plant interactions, driving plant growth promotion and the biological control of plant pathogens. The application of AHLs or synthetic consortia incorporating AHL-producing plant growth-promoting bacteria to plants for priming plant-beneficial activities may offer significant opportunities for improving crop yields [68].

Given the enormous threat to human and animal health posed by multi-antibiotic-resistant bacteria and biofilm-associated infections worldwide, alternative therapeutic approaches are required to prevent or treat bacterial infections. In this context, the attenuation of bacterial virulence and biofilm formation has been viewed as attractive targets for the development of ‘anti-virulence’ agents that could be used individually or as antibiotic ‘adjuvants’ via combination therapy [69]. For most pathogens, virulence is multifactorial and relies on the expression of diverse cell-associated and secreted virulence determinants and the ability to form surface-associated antibiotic-tolerant, host-defence-resistant biofilms. In common human pathogens such as *P. aeruginosa* and *S. aureus*, targeting QS offers opportunities to inhibit the expression of multiple virulence factor genes and render biofilms susceptible to antibiotics [30, 69, 70].

QS systems provide multiple druggable molecular targets for inhibitors for quorum sensing inhibitors (QSIs) that include the enzymes involved in QS signal molecule biosynthesis and the receptors involved in signal transduction [70, 71]. Considerable advances in our understanding of the chemical biology of QS systems and their inhibition have been made, some promising QS targets structurally characterized, QSI screens developed and inhibitors identified [70, 71]. However, although QSIs remain a promising avenue for anti-virulence treatment, their translation faces notable challenges. While most QSIs identified in the literature show efficacy *in vitro*, many are likely pan-assay interference compounds that fail to demonstrate direct interaction(s) with their target [72]. Others exhibit only moderate potency, high lipophilicity, poor bioavailability, metabolic (and chemical) instability and cytotoxicity in human cell lines [73, 74]. Many have failed or given highly variable results when tested against clinical isolates or in biofilms, especially in polymicrobial contexts or through bacterial adaptation to repeated treatment [63, 75, 76]. An additional challenge is that some inhibitors, and even native QS signals, can be exported via multidrug efflux pumps, lowering intracellular exposure [77].

It remains to be experimentally demonstrated why so many pathogens link virulence factor production to cell density-dependent QS. In some contexts, delayed expression may prevent premature host detection, ensuring that energetically expensive secreted products reach effective concentrations to coordinate a collective attack once a sufficient infectious burden is present or couple virulence to host-associated microenvironments. However, these explanations remain incompletely tested across pathogens and infection models. This uncertainty is directly relevant to anti-QS therapy: if QS-dependent virulence provides context-specific benefits, successful intervention will require identifying when and where disrupting communication reduces disease without simply selecting for regulatory diversion and activation of compensatory virulence pathways.

Therefore, QSIs which demonstrate robust performance in physiologically relevant multispecies and host-associated environments bypassing the limitations indicated above are required. Future work must determine how QSIs behave in complex microbiomes, evaluate their long-term evolutionary robustness, optimize combined QSI-antibiotic regimens and establish standardized *in vivo* models that mimic realistic polymicrobial infections in the host. Thus, much more work is required before any QSI ‘hits’ with the appropriate pharmacological and pharmacokinetic properties can enter human clinical trials. Indeed, the relative efficacy and potency of QSIs alone or as prophylactics or therapeutics or as adjuvants in combination with conventional antibiotics still needs to be extensively evaluated *in vivo*. Specific attention needs to be given to the measurement of successful QSI therapy outcomes with respect to bacterial clearance, immune response and pathophysiology. Currently, our understanding of the potential of QS as an antibacterial target suggests that it is likely to be of limited value.

## Conclusions

QS is neither a simple density switch nor a closed bacterial conversation, but a context-dependent, evolving network shaped by space, hosts and communities. Addressing these knowledge gaps will require, among other efforts, quantitative *in situ* measurements, high-throughput discovery of non-canonical chemistries and models that capture polymicrobial dynamics and evolutionary trajectories. Integrating microbiology, chemical biology, biophysics and clinical ecology will clarify when interference dominates, which thresholds matter and which nodes are druggable, thereby enabling robust, evolution-informed anti-virulence strategies that translate beyond the laboratory flask. Thus, the original emphasis on ‘quorum’ may no longer fully capture the nature of bacterial collective behaviour, which depends not only on cell number but also on the unity and coordination of the population response; as the American revolutionary and writer Thomas Paine observed, ‘It is not in numbers, but in unity, that our great strength lies.’

## Abbreviations

ACLs: *N*-acyl-cyclolysines
AHLs: *N*-acylhomoserine lactones
AI-2: autoinducer-2
AQ: 2-alkyl-4-quinolone
QS: quorum sensing
QSIs: quorum sensing inhibitors
